# Impact of DBBM Fragments on the Porosity of the Calvarial Bone: A Pilot Study on Mice

**DOI:** 10.3390/ma13214748

**Published:** 2020-10-23

**Authors:** Ulrike Kuchler, Patrick Heimel, Alexandra Stähli, Franz Josef Strauss, Bernadette Luza, Reinhard Gruber

**Affiliations:** 1 Department of Oral Surgery, University Clinic of Dentistry, Medical University of Vienna, 1090 Vienna, Austria; ulrike.kuchler@meduniwien.ac.at; 2 Core Facility Hard Tissue and Biomaterial Research, Karl Donath Laboratory, University Clinic of Dentistry, Medical University of Vienna, 1090 Vienna, Austria; patrick.heimel@trauma.lbg.ac.at (P.H.); bernadette.luza@gmx.at (B.L.); 3 Ludwig Boltzmann Institute for Clinical and Experimental Traumatology, 1090 Vienna, Austria; 4 Austrian Cluster for Tissue Regeneration, 1090 Vienna, Austria; 5 Department of Oral Biology, University Clinic of Dentistry, Medical University of Vienna, 1090 Vienna, Austria; alexandra.staehli@zmk.unibe.ch (A.S.); franz.strauss@zzm.uzh.ch (F.J.S.); 6 Department of Periodontology, School of Dental Medicine, University of Bern, 3010 Bern, Switzerland; 7 Department of Conservative Dentistry, School of Dentistry, University of Chile, Santiago 8380544, Chile; 8 Clinic of Reconstructive Dentistry, Center of Dental Medicine, University of Zurich, 8032 Zurich, Switzerland

**Keywords:** bone augmentation, guided bone regeneration, osteoclast, biomaterial, DBBM, mouse, resorption, calvarial bone

## Abstract

Deproteinized bovine bone mineral (DBBM) is brittle and can break into fragments. Here, we examined whether DBBM fragments have an impact on mice calvarial bone during bone augmentation. DBBM was either randomly crushed (DBBM fragments) or left undisturbed (DBBM granules). Then, DBBM fragments or original DBBM granules were placed onto calvarial bone in 20 BALB/c mice. Following random allocation, ten mice received DBBM fragments and ten mice received original DBBM granules. After fourteen days of healing, micro computed tomography (micro-CT) and histological analysis of the augmented sites were performed. The primary outcome was the porosity of the calvarial bone. The micro-CT analysis revealed that DBBM fragments failed to significantly change the porosity of the calvarial bone as compared with original DBBM granules, despite the slightly higher bone resorption in the DBBM fragment group, 10.3% (CI 6.3–11.6) versus 6.1% (CI 4.1–7.8, *p* = 0.355), respectively. The cortical bone volume was not altered by DBBM fragments as compared with original DBBM granules, i.e., 79.0% (CI 78.9–81.2) versus 81.5% (CI 80.1–83.3, *p* = 0.357), respectively. The DBBM fragment group revealed similar bone thickness values as compared with the DBBM granules group, i.e., 0.26 mm (CI 0.23–0.29) versus 0.25 mm (CI 0.22–0.27, *p* = 0.641), respectively. The histological evaluation supported the micro-CT observations, displaying minor signs of porosity and resorption. The particle-size distribution analysis confirmed a shift towards smaller particle sizes in the DBBM fragment group. These findings suggest that DBBM fragments behave similarly to original DBBM granules in terms of bone morphological changes at augmented sites.

## 1. Introduction

Bone substitutes are widely used in regenerative dentistry, mainly to avoid the harvesting of autografts prior to augmentation procedures. Unlike bone autografts, certain bone substitutes can resist resorption, and thereby can maintain the augmented volume [1,2]. Bone substitutes are generally osteoconductive, providing a surface and guidance for the newly formed bone within the augmented site [3]. This newly formed and immature bone, known as woven bone, is later reinforced and remodeled into a mature lamellar bone [4]. While transient inflammation is required for bone regeneration [5], chronic inflammation causes osteolysis [6] and suppresses bone formation [7]. Consequently, under uncontrolled inflammatory conditions, the overall process of graft consolidation can be compromised. Therefore, it becomes critical to understand the host–biomaterial interaction at augmented sites.

Inflammation is usually initiated by blood coagulation. Once microbial cues such as endotoxins or damaged cells are removed, inflammation resolves, and wound healing and, subsequently, bone regeneration can proceed [8]. Therefore, bone substitutes should not elicit a chronic inflammatory response, but sometimes they do. For example, wear particles released from hip prostheses can induce an aseptic inflammation causing osteolysis and fibrous encapsulation of the non-degraded biomaterial [9]. Moreover, small hydroxyapatite particles (<20 μm) induce stronger inflammatory responses as compared with larger sized particles [10]. On a cellular level, macrophages are activated by the non-digestible microparticles they engulf or by frustrated macrophages forming multinucleated giant cells [11,12]. Since bone substitute materials can release small particles during graft consolidation, it is conceivable that these small particles could have an impact on bone remodeling and, consequently, on the integrity of calvarial bone.

Deproteinized bovine bone mineral (DBBM) is a widely used bone substitute in regenerative dentistry [13]. DBBM is osteoconductive and there are manifold clinical indications including small and large bone augmentations [14]. DBBM, however, is brittle and can break into smaller fragments [15]. Considering that small DBBM fragments attract more mono- and multinucleated giant cells as compared with regular DBBM granules [16], it can be hypothesized that small DBBM fragments could be recognized by the local macrophages affecting local bone remodeling. On the basis of a particle-induced osteolysis model in which cortical porosity is increased in response to polyethylene particles and endotoxins [17,18,19], the aim of the present study was to examine the morphological changes of calvarial bone augmented either with DBBM fragments or regular DBBM granules.

## 2. Results

### 2.1. Micro-CT and Histological Analysis Following Bone Augmentation with Regular Deproteinized Bovine Bone Mineral (DBBM) Granules and DBBM Fragments

**Figure 1:**
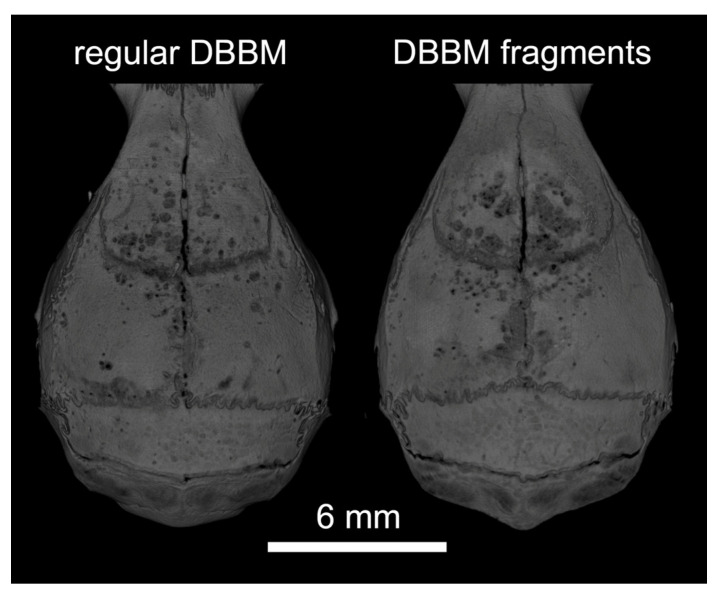
Representative micro-CT images of both groups at the augmented area after 14 days of healing. The calvarial bone was augmented either with regular DBBM granules or DBBM fragments. A few signs of resorption are noticeable on the bone surface in both groups.

**Figure 2:**
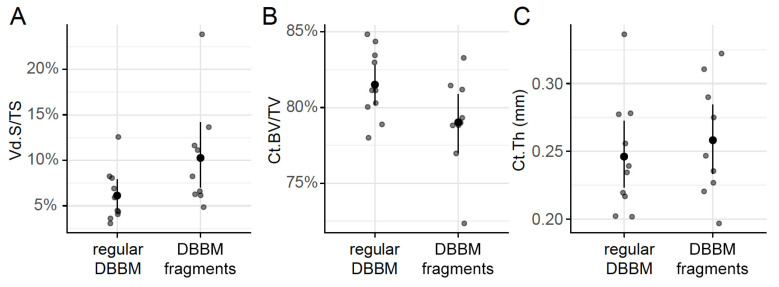
Micro-CT analysis of the calvarial bone. Cortical porosity (**A**) (void surface/tissue surface, Vd.S/TS in %), cortical bone volume/tissue volume (**B**) (Ct.BV/TV in %), and cortical thickness (**C**) (Ct.Th in mm) were determined based on micro-CT data. The data are presented using scatterplots with a mean and a corresponding 95% confidence interval. No significant differences were noticed between the groups. Statistical analysis was based on ANOVA permutation test.

**Figure 3:**
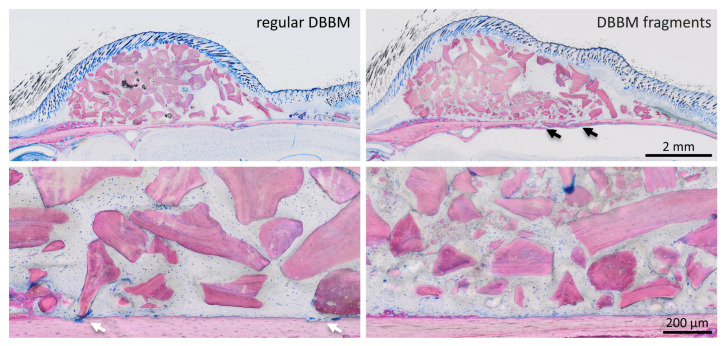
Histological analysis of calvarial bone. Representative undecalcified thin-ground sections were stained with Levai–Laczko dye, parallel to the sagittal suture and through the center of the augmented area. The higher magnification clearly shows the different size of DBBM particles (light purple) between the groups. The periosteal surface of calvarial bone (light pink) revealed minimal signs of resorption with a slight trend towards more bone resorption (black arrows) in the DBBM fragment group. In the regular DBBM, a few multinucleated cells, presumably osteoclasts, are visible (white arrows) causing a weak resorption.

Two mice died during surgery, and as a result, a total of 18 mice were analyzed (Supplementary Figure S1). Micro-CT analysis revealed a few and similar signs of bone resorption in both groups (Figure 1). Quantitative analysis showed that, with regular DBBM, the void surface of the calvarial bone, indicating the cortical porosity, was 6.1% (CI 4.1–7.8), and with DBBM fragments it was somewhat higher, despite not reaching statistical significance with 10.3% (CI 6.3–11.6, *p* = 0.355) (Figure 2A). In support of these observations, cortical bone volume (BV/TV) was 81.5% (CI 80.1–83.3) and 79.0% (CI 78.9–81.2, *p* = 0.357) with regular and DBBM fragments, respectively (Figure 2B). The mean thickness of the cortical bone was 0.25 mm (CI 0.22–0.27) in the regular DBBM group and 0.26 mm (CI 0.23–0.29) *p* = 0.641) in the DBBM fragment group. The histological evaluation supported the micro-CT results. As indicated in Figure 3, regular DBBM granules and DBBM fragments displayed comparable small resorption sites and moderate new bone formation on the endocranial surface of the calvarial bone.

### 2.2. Particle-Size Distribution and Histological Analysis of Regular DBBM Granules and DBBM Fragments

**Figure 4:**
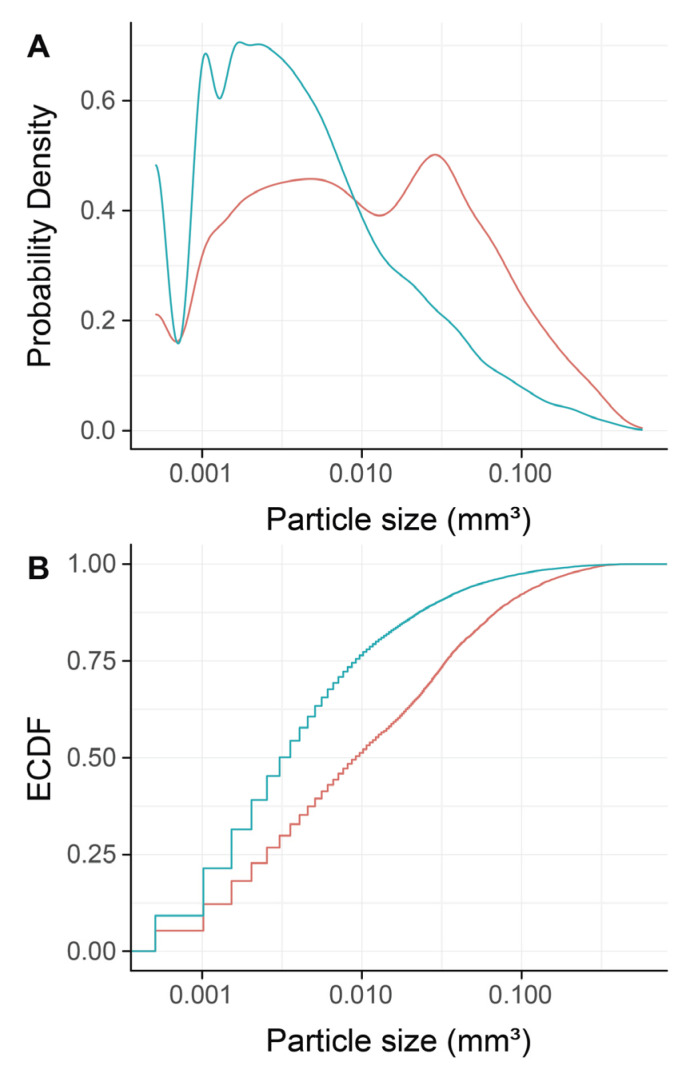
Micro-CT analysis of deproteinized bovine bone mineral (DBBM), size distribution of DBBM fragments. Empirical cumulative probability functions (**A**,**B**) for the particle size (log scale). The statistical analysis showed significant differences in the size distribution of the DBBM fragments (turquois) as compared with regular DBBM granules (red) (*p* < 0.001). The statistical analysis was based on ANOVA permutation test.

**Figure 5:**
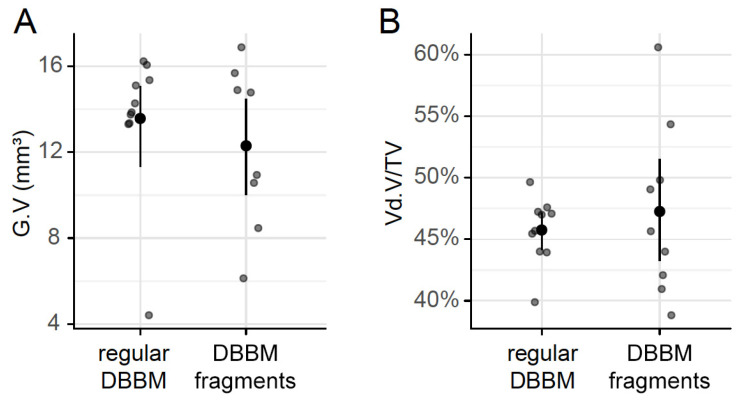
Micro-CT analysis of DBBM. The total graft volume (**A**) (G.V in mm^3^) and the porosity of the augmented area (**B**) (void volume/tissue volume, Vd.V/TV in %) showed no changes between regular DBBM granules and DBBM fragments. The data are presented using scatterplots with mean and a corresponding 95% confidence interval. No significant differences were noticed between the groups. The statistical analysis was based on ANOVA permutation test.

To confirm that DBBM fragments had a smaller size than regular DBBM granules, a segmentation of the particles was carried out. The particle-size distribution of the DBBM particles ranged from 0.001 mm^3^ to more than 0.1 mm^3^, and there were significant differences (*p* < 0.001) when regular DBBM granules and DBBM fragments were compared (Figure 4A,B). Micro-CT analysis further showed a relative graft volume (G.V.) of 13.57 mm^3^ (CI 13.45–15.29) and 12.30 mm^3^ (CI 10.57 max 14.89, *p* = 0.057) in the regular DBBM granules group and in the DBBM fragmented group, respectively (Figure 5A). In addition, the space between the DBBM particles was comparable between the groups as indicated by the porosity of the augmented area (Figure 5B). The porosity of the augmented area (void volume/tissue volume, Vd.V/TV in %) was 45.8% (CI 44.4–47.2) in the regular DBBM group and 47.3% (CI 42.1– 49.8, *p* = 0.092) in the DBBM fragmented group. The DBBM fragments failed to noticeably change the presence of macrophages or osteoclast-like cells (Figure 3).

## 3. Discussion

The present study revealed comparable morphological changes in the cortical bone between regular DBBM granules and DBBM fragments. Although DBBM fragments showed a trend for a higher bone porosity, the cortical bone volume and the respective thickness of the cortex were rather similar between the two groups. Overall, these findings indicate that smaller DBBM fragments behave similarly to regular DBBM granules.

The present results are in line with a recent preclinical study where two different DBBM particle sizes were compared. Through a sinus augmentation model with simultaneous implant placement, the study revealed similar amounts of bone regeneration between small and large DBBM particles [20]. In the present study, however, the preparation of small DBBM particles was performed by crushing regular DBBM particles in order to simulate a random distribution of smaller but also larger DBBM particles. The calvarial augmentation model is an established setting to identify possible morphological bone changes under inflammatory conditions. On the basis of this model, a recent preclinical study revealed that lipopolysaccharides (LPS) and polyethylene particles provoked a robust osteolysis with a strong compensatory production of woven bone, indicated by a high cortical porosity of 21% and 14%, respectively [17]. These previous preclinical data support the use of the present model to study the integrity and the morphological changes of calvarial bone using DBBM fragments.

Considering the character of a pilot proof-of-principle study, the clinical relevance of this strategy has to be interpreted with caution. The calvarial model does not effectively represent the majority of clinical scenarios, where the grafted bone particles are placed onto well-bleeding recipient beds. In the current model, the DBBM was placed on an intact cortical bone surface with a limited osteogenic potential [21]. Moreover, the grafted granules and fragments are mechanically better protected by barrier membranes as opposed to the current setting where the particles were in direct contact with the overlying periosteum and skin, providing minimal mechanical protection. It should be mentioned, however, that the displacement of the bone substitutes could not completely be avoided despite the presence of barrier membranes [22,23]. Furthermore, there was a large variation in the parameters measured within both groups. This suggests that the model may not be capable of detecting subtle changes in bone remodeling. Given the single time point chosen [17], the response of the calvarial bone at longer healing periods remains to be determined. In addition, there are new materials, including tricalcium phosphate bioceramics [24] with a controllable degradation, that may react differently, but were not evaluated in the present study. These limitations should be considered when interpreting the overall positive findings of this pilot study.

Instead of using DBBM fragments with a defined size, the present study reported the size distribution of the particles. As expected, the size distribution analysis revealed DBBM fragments, as well as original size DBBM granules. In this sense, it cannot be ruled out that DBBM fragments smaller than those observed in the present report could indeed affect the integrity of the cortical bone in this model. In addition, the spatial distribution of the different size fragments could not be controlled and this could have influenced a given response. Considering that no signs of an ongoing inflammation were detected in the histology, the establishment of an inflammatory score was not possible. In this regard, we had to rely on our previous findings using an LPS-induced calvarial osteolysis mice model. In that sister study, LPS-induced osteolysis caused a massive increase in cortical porosity with compensatory woven bone formation [17].

Taken together and within the limitations of the current proof-of-principle study, these findings suggest that DBBM fragments do not impair bone remodeling at augmented sites implicating a similar behavior to regular DBBM granules in a rat model.

## 4. Material and Methods

### 4.1. Study Design

The study protocol was approved by the ethical review board for animal research of the Medical University of Vienna (GZ BMWFW-66.009/0193-WF/V/3b/2016). The study was performed in 2017 at the Department of Biomedical Research of the Medical University of Vienna in accordance with the ARRIVE guidelines. Twenty BALB/c mice (8–10 weeks, 20–25 g) from the Division for Biomedical Research (Himberg, Austria) were randomly divided into two groups with 10 animals each, based on a computer-generated list.

### 4.2. Calvarial Osteolysis Model

Ulrike Kuchler, Franz Josef Strauss, and Alexandra Stähli performed the surgeries. All animals received i.m. ketamine 100 mg/kg (AniMedica, Senden, Erlangen, Germany) and xylazine hydrochloride 5 mg/kg (Bayer Austria, Vienna, Austria). After an incision over the calvaria, the periosteum was elevated, and the space filled either with regular DBBM or DBBM fragments that had been crushed with a standard hammer. The wounds were closed with resorbable sutures (Vicryl 6-0, Ethicon GmbH, Norderstedt, Germany). For pain relief, buprenorphine 0.06 mg/kg s.c. (Temgesic^®^, Temgesic, Reckitt and Colman Pharm., Hull, UK) and piritramide in drinking water ad libitum was administered. At day fourteen, the calvarial samples were subjected to micro-computed tomographic (micro-CT) and histological analysis.

### 4.3. Micro-CT Analysis

Calvarial samples were fixed in phosphate-buffered formalin (Roti-Histofix 4%, Carl Roth, Karlsruhe, Germany). The µCT images, taken at 90 kV/200 µA, isotropic resolution of 17.2 µm and an integration time of 500 ms (µCT 50, SCANCO Medical AG, Bruttisellen, Switzerland), were rotated using Amira 6.2 (Thermo Fisher Scientific, Waltham, MA, USA). The images underwent rigid registration so that the samples were equally positioned and oriented in all scans. The region of interest (ROI) was defined comprising the augmented compartments of the defect area (Figure 1). Using the Definiens Developer XD2^®^ software (Munich, Germany, Version 2.1.1), the ROIs were positioned manually and automatically segmented from the µCT images. At the interior surface of the calvarial bone, we determined the cortical porosity (void surface/tissue surface, Ct.Vd.S/TS in %), cortical bone volume/tissue volume (Ct.BV/TV in %), and cortical thickness (Ct.Th, mm). The DBBM particle size distribution was measured. In addition, the total DBBM graft volume (mm^3^) and the relative proportion of soft tissue in the augmented site (void volume/tissue volume, Vd.V/TV in %) were determined. Measurements and calculations were performed with the Definiens Developer XD2^®^ software (Munich, Germany, Version 2.1.1). Calibration and blinding of the examiner were not necessary as the software performed the analysis automatically and identically for all samples. Further details are reported elsewhere [17].

### 4.4. Histological Analysis

Calvarial samples were dehydrated with ascending alcohol grades and embedded in light-curing resin (Technovit 7200 VLC + BPO, Kulzer & Co., Wehrheim, Germany). Blocks were further processed using Exakt cutting and grinding equipment (Exakt Apparatebau, Norderstedt, Germany). Thin-ground sections, from all samples, were prepared and stained with Levai–Laczko dye in a plane parallel to the sagittal suture and through the center of the augmented area. The slices were scanned using an Olympus BX61VS digital virtual microscopy system (DotSlide 2.4, Olympus, Japan, Tokyo) with a 20× objective resulting in a resolution of 0.32 µm per pixel, and then evaluated.

### 4.5. Statistics

The data are presented graphically using scatterplots, overplotted by the mean and a corresponding bootstrap 95% confidence interval (CI). A non-parametric approach was used for inference as follows: In the first step, an ANOVA-type permutation test was calculated based on B = 10,000. In the case of significance, pairwise post hoc permutation tests were performed, using the Step-down maxT procedure to account for multiple testing [25,26]. All computations were done using R version 3.5.1 (R Core Team 2018, Vienna, Austria). Owing to the pilot nature of the study, the sample size was chosen based on experience from previous studies [27] to balance the ability to measure significant differences while reducing the number of animals needed.

## Supplementary Materials

The following are available online at https://www.mdpi.com/1996-1944/13/21/4748/s1, Figure S1: All samples overview.
