# Supplementary material for: Impact of DBBM Fragments on the Porosity of the Calvarial Bone: A Pilot Study on Mice
Source: Materials (Basel). 2020 Oct 23;13(21):4748. doi: 10.3390/ma13214748 (PMC7660694; doi:10.3390/ma13214748)
Supplement: Supplementary file 1 [file materials-13-04748-s001.pdf]

materials

MDPI

Supplementary material

Impact of DBBM Fragments on the Porosity of the Calvarial Bone: A Pilot Study on Mice

Ulrike Kuchler [1], Patrick Heimel [2,3,4], Alexandra Stähli [5,6], Franz Josef Strauss [5,7,8], Bernadette Luza [2,3] and Reinhard Gruber [5,6,*]

1. Department of Oral Surgery, University Clinic of Dentistry, Medical University of Vienna, 1090 Vienna, Austria; ulrike.kuchler@meduniwien.ac.at
2. Core Facility Hard Tissue and Biomaterial Research, Karl Donath Laboratory, University Clinic of Dentistry, Medical University of Vienna, 1090 Vienna, Austria; patrick.heimel@trauma.lbg.ac.at (P.H.); bernadette.luza@gmx.at (B.L.)
3. Ludwig Boltzmann Institute for Clinical and Experimental Traumatology, 1090 Vienna, Austria
4. Austrian Cluster for Tissue Regeneration, 1090 Vienna, Austria
5. Department of Oral Biology, University Clinic of Dentistry, Medical University of Vienna, 1090 Vienna, Austria; franz.strauss@zzm.uzh.ch
6. Department of Periodontology, School of Dental Medicine, University of Bern, 3010 Bern, Switzerland; alexandra.staehli@zmk.unibe.ch
7. Department of Conservative Dentistry, School of Dentistry, 8380544, University of Chile, Santiago, Chile
8. Clinic of Reconstructive Dentistry, Center of Dental Medicine, University of Zurich, 8032 Zurich, Switzerland.

* Correspondence: reinhard.gruber@meduniwien.ac.at, Tel.: +43-699-107-18-472

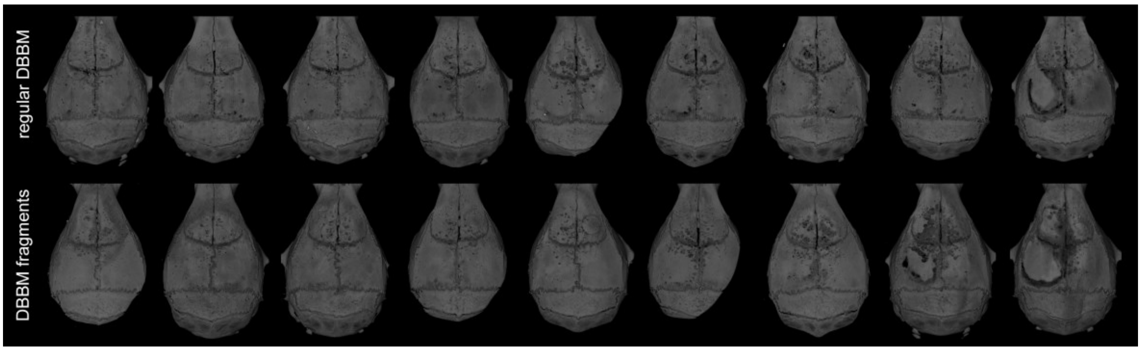

Figure S1. All samples overview.
